# Web Search Behavior and Information Needs of People With Multiple Sclerosis: Focus Group Study and Analysis of Online Postings

**DOI:** 10.2196/ijmr.3034

**Published:** 2014-07-24

**Authors:** Cinzia Colombo, Paola Mosconi, Paolo Confalonieri, Isabella Baroni, Silvia Traversa, Sophie J Hill, Anneliese J Synnot, Nadia Oprandi, Graziella Filippini

**Affiliations:** ^1^IRCCS-Mario Negri Institute for Pharmacological Research, Milano, ItalyDepartment of Public Health, Laboratory for medical research and consumer involvementMilanoItaly; ^2^IRCCS FoundationMultiple Sclerosis CenterMilanoItaly; ^3^Italian Multiple Sclerosis FoundationGenovaItaly; ^4^Centre for Health Communication and ParticipationDepartment of Public Health, School of Public Health and Human BioscienceLa Trobe UniversityMelbourneAustralia; ^5^SistemikaPadovaItaly

**Keywords:** multiple sclerosis, evidence-based information, information needs, Web search behavior, Internet, patients’ involvement

## Abstract

**Background:**

Multiple sclerosis (MS) patients and their family members increasingly seek health information on the Internet. There has been little exploration of how MS patients integrate health information with their needs, preferences, and values for decision making. The INtegrating and Deriving Evidence, Experiences, and Preferences (IN-DEEP) project is a collaboration between Italian and Australian researchers and MS patients, aimed to make high-quality evidence accessible and meaningful to MS patients and families, developing a Web-based resource of evidence-based information starting from their information needs.

**Objective:**

The objective of this study was to analyze MS patients and their family members’ experience about the Web-based health information, to evaluate how they asses this information, and how they integrate health information with personal values.

**Methods:**

We organized 6 focus groups, 3 with MS patients and 3 with family members, in the Northern, Central, and Southern parts of Italy (April-June 2011). They included 40 MS patients aged between 18 and 60, diagnosed as having MS at least 3 months earlier, and 20 family members aged 18 and over, being relatives of a person with at least a 3-months MS diagnosis. The focus groups were audio-recorded and transcribed verbatim (Atlas software, V 6.0). Data were analyzed from a conceptual point of view through a coding system. An online forum was hosted by the Italian MS society on its Web platform to widen the collection of information. Nine questions were posted covering searching behavior, use of Web-based information, truthfulness of Web information. At the end, posts were downloaded and transcribed.

**Results:**

Information needs covered a comprehensive communication of diagnosis, prognosis, and adverse events of treatments, MS causes or risk factors, new drugs, practical, and lifestyle-related information. The Internet is considered useful by MS patients, however, at the beginning or in a later stage of the disease a refusal to actively search for information could occur. Participants used to search on the Web before or after their neurologist’s visit or when a new therapy was proposed. Social networks are widely used to read others’ stories and retrieve information about daily management. A critical issue was the difficulty of recognizing reliable information on the Web. Many sources were used but the neurologist was mostly the final source of treatment decisions.

**Conclusions:**

MS patients used the Internet as a tool to integrate information about the illness. Information needs covered a wide spectrum, the searched topics changed with progression of the disease. Criteria for evaluating Internet accuracy and credibility of information were often lacking or generic. This may limit the empowerment of patients in health care choices.

## Introduction

Providing health care information and tackling the right questions at the right time together with professional advice can improve people’s knowledge of the disease, reduce anxiety, facilitate symptom management, and increase a sense of empowerment [1]. In multiple sclerosis (MS), giving newly-diagnosed people targeted information improves their knowledge of MS and satisfaction with care [2,3]. With the advent of disease-modifying drugs, MS patients increasingly seek information about new treatments, and earlier attitudes of hopelessness have changed [4]. In general, more and more people demand active roles in medical decision-making and asking for health and research information to share decisions with doctors about treatment and management options [5,6]. They want to know the evidence behind different treatments [7], how research relates to them [8], and the implications of research findings for their health care options and choices. This creates a challenge for providing health information based on research and connections between the research and individuals, to enable people to apply research findings to their own circumstances. There are two good reasons to catch patients’ information needs. First, to make relevant information available to patients, a research governance strategy bringing together researchers and patients is needed [9]. Second, a definite judgment of treatment effect needs to incorporate patient’s voice into the design of the therapeutic programs [10].

Research-based health information has become the topic of studies focusing on how to present it clearly and unambiguously [7,11]. The most accessible and usable formats to communicate research-based information have also been studied [12-14].

One of the main sources of health care information is the Internet. According to recent surveys in the United Kingdom and Canada, it is now placed second to health professionals as a source [7]. Approximately 70% of individuals in the European Union use the Internet and almost 40% of those aged 16 to 74 use it to seek health information [15]. In Italy, almost one-half of people use the Internet, and more than a one-quarter of those aged 16 to 74 use it to seek health information [15,16]. The strongest users are aged 11 to 34 years.

MS patients, like other people with chronic conditions, increasingly search for health information on the Internet [17,18], also using YouTube, Facebook, blogs, or forums. The use of Web 2.0 as a source of information on new controversial treatments raised debate about its role, both in personal decision-making and in public demand for health care services or interventions [19,20].

To assess the accuracy of health information and use it to exert greater control over life events and situations, critical appraisal skills are essential [21]. This is particularly true for Web-based information, where skill is needed to judge the degree to which the information merits trust accordingly with the evidence available [22]. Patients’ associations have a critical role in giving information to MS patients and family members [23] and increasingly use websites and social networks to provide information and refer people to high-quality sources. This reflects the need to encourage skilled, confident information users and to promote a higher level of patients’ and community engagement in health care.

Health care and service providers can take different roles in relation to Internet information-seeking behavior [24]: working in partnership to obtain and analyze information, guiding patients in finding reliable sites, or dismissing patients’ information queries. The INtegrating and Deriving Evidence, Experiences and Preferences (IN-DEEP) project, aligns itself with the first two roles. It is a collaboration between research teams in Italy and Australia, developing two parallel projects following the same steps and a mixed-methods approach. The projects involve researchers in health communication, neurologists, MS patients, MS patients’ associations (MS Australia and Italian Multiple Sclerosis Association [AISM]). This project is focused on Web-based health information with the aim to make high-quality evidence more accessible and meaningful to MS patients and their families, in particular, starting from their information needs, to develop a Web-based source of evidence-based health information. A four-stage process has been developed: first the assessment of health information needs through qualitative research, second the development of a Web template for presenting evidence-based health information, third the implementation of a pilot Internet template, and fourth a Web-based survey to evaluate if the IN-DEEP Web-based resource meets the information needs of MS patients and family members. [25]

The present article deals with the first qualitative stage, aimed at documenting and analyzing MS patients’ and families’ experience in finding, assessing, integrating Web-based health information with personal values.

## Methods

### Protocol

The IN-DEEP protocol was published [25] and the ethical approval has been granted by the Faculty of Health Sciences Human Research Ethics Committee of La Trobe University, Australia, and the Ethics Committee of the Fondazione Istituto di Ricovero e Cura a Carattere Scientifico Istituto Neurologico “Carlo Besta,” Italy. Face-to-face focus groups were formed with MS patients and family members and a Web-based forum was also proposed as an additional method to involve more people using the Internet and widen the collection of information.

### Focus Groups

We organized 6 focus groups, 3 with MS patients and 3 with family members, in Milan (North), Macerata (Center), and Palermo (South) from April to June 2011. MS patients aged between 18 and 60, diagnosed as having MS at least 3 months earlier, according to the Poser et al [26] or McDonald diagnostic criteria [27,28], and using the Web to search for information on MS were included.

For family members, inclusion criteria were age 18 and over, being a relative of a person with at least a 3-months MS diagnosis and using the Web to seek information on MS. The period of 3-months was an arbitrary choice aimed to include new MS people but not so close to the diagnosis, leaving a certain period of time to experience Web searching about MS. Family members of MS patients who took part in focus groups were excluded to increase the variety of sources, and make people feel guaranteed in sharing their opinion with no fear to be contradicted. MS patients and family members were invited by a group of neurologists and by AISM local units.

A purposeful sampling approach was used to select the participants. A screening questionnaire (SQ) completed in the presence of neurologists was used to collect information about participants. SQ was adopted to keep the project team in control of the recruitment process, maximizing the internal variability among participants and assuring they meet the including criteria at the same time. The SQ items covered sociodemographic characteristics and information about MS (year of diagnosis, type, Expanded Disability Status Scale (EDSS; as a standardized method of quantifying disability in MS, and therapy; Multimedia Appendix 1). Some questions focused on the frequency of Internet use and the kind of information sought. Selection was designed to obtain the most balanced sample in terms of age, education, MS length and severity, and a female/male ratio of 3:1. The focus groups were led by an expert moderator and an assistant moderator, using an interview guide as an outline for the focus group (Multimedia Appendix 2).

### Online Forum

The AISM hosted the Web-based forum on its Web platform for 1 month. Nine questions, and four subquestions, were posted covering searching behavior, use of Web-based information, truthfulness of Web information (Multimedia Appendix 3). AISM invited MS patients and family members by email and through the website. Participants received an information sheet by email, signed a consent form, and completed the SQ, as described above. Once the consent and SQ were returned to the researchers, they gave each participant a predefined username and password. Researchers were given moderator rights to post comments, to stimulate discussion, and assist participants. At the end, they downloaded a transcript of the forum, AISM closed the forum, and deleted the data.

### Data Analysis

Focus groups were audio-recorded and transcribed verbatim. Atlas.ti software (V 6.0) was used [29,30]. Data were analyzed from a conceptual point of view through a coding system [31]. The moderator and the assistant moderator independently read several times the transcripts and agreed on a common set of codes (ie, labels corresponding to emerging concepts). The analysis was guided by the research questions (see the interview guide, Multimedia Appendix 2) and the results were summarized according to them. Findings from focus group were analyzed together with data collected through the online forum.

## Results

### Participant Characteristics

In total 40 MS patients and 20 family members (mother/father 6, wife/husband 9, sister/brother 1, daughter/son 4) participated in the focus groups and the online forum (Table 1). All the MS patients, except 1, used the Internet for gathering information, 37 of them declared that they used it for health purposes.

**Table 1:** Participants’ main characteristics.^a^

		Focus groups			Online forum		
		All participants n=41	People with MS n=24	Family member^b^ n=17	All participants n=19	People with MS n=16	Family member^b^ n=3
**Gender, n (%)**							
	Female	26 (63)	17 (71)	9 (53)	16 (84)	14 (88)	2 (67)
	Male	15 (37)	7 (29)	8 (47)	3 (16)	2 (13)	1 (33)
**Age, years**							
	Mean (SD)	41.6 (10.3)	40.5 (10.2)	43.3 (10.6)	46.2 (10.9)	44.9 (11.4)	53.3 (2.9)
	Median	42.0	42.0	41.0	46.0	42.5	55.0
	Range	24-66	27-57	31-66	27-67	27-67	50-55
**Internet use for health purposes, n (%)**							
	Yes	37 (97)	24 (100)	14 (93)	19 (100)	16 (100)	3 (100)
	No	1 (3)	0 (0.00)	1 (7)	0 (0)	0 (0)	0 (0)
**Education, n (%)**							
	Primary school	8 (20)	4 (17)	4 (25)	1 (5)	1 (6)	0 (0)
	Italian Middle school	4 (10)	2 (8)	2 (12)	2 (11)	2 (13)	0 (0)
	Secondary school	18 (45)	9 (38)	9 (56)	9 (47)	7 (44)	2 (67)
	University	10 (25)	9 (38)	1 (6)	7 (37)	6 (38)	1 (33)
**Years with MS** ^c^							
	Mean (SD)	11.0 (9.4)	9.1 (7.6)	13.9 (11.4)	12.2 (9.9)	10.6 (8.8)	20.7 (13.4)
	Median	9.5	9.0	9.5	10.0	9.5	15.0
	Range	0-33	0-32	1-33	2-36	2-31	11-36
**EDSS** ^d^							
	Median	2.5	2.5	4.0	3.0	3.0	5.0
	Range	0-8	0-7	0-8	1-9	1-9	3-7

^a^Some discrepancies in the total are due to some missing values.

^b^Years with MS, and EDSS reported in family members columns is related to the MS patient cared by a family member

^c^Multiple sclerosis.

^d^Expanded Disability Status Scale.

### Information Needs

Main information needs of MS patients and family members were about treatments, symptoms’ management, and causes of the illness:

When I was first diagnosed, I trawled the Internet looking for information on the disease, its biological mechanisms, and how to reverse symptoms. I often used Wikipedia to have a rough idea of the nervous system functioning and how the disease changes it.

MS patient, focus group

Among main interests was information about drugs, those they are using or those suggested by the neurologist, and adverse effects, new drugs or treatments available or under trial also abroad, how and where to have access to tests and new treatments, alternative therapies and diet. One of the most widely searched topics was new treatments. An example cited by participants was chronic cerebrospinal venous insufficiency (CCSVI). This condition, brought to the forefront by television programs, was looked for by the participants, and a few of them acted independently from their neurologist’s opinion by doing or booking the echo-color Doppler and the percutaneous transluminal angioplasty. As reported by a participant: “I consulted the Internet and clarified my ideas, because the medical Professor, as you have probably seen on you tube, was very clear” [MS patients, focus group]; “For example, when there had been the congress in Goteborg, there were the abstracts of all the presentations … so one could have read what had been actually said” [MS patient, focus group].

The online forum participants searched for suggestions and information on how to cope with everyday life, managing symptoms and problems due to MS or drugs, such as fatigue, bladder disturbances, how to have a satisfactory sex life despite disability, how to avoid unsightly bruising due to interferon injections. Participants in the online forum admitted: “Being protected by the anonymity of the Internet, it is easier to look for information/exchanges with other patients on sensitive topics, like bladder problems or related to sex” [MS patient, online forum]. Life habits, as smoking, alcohol use, attending gym, etc, and their relation with MS were topics of interest, especially among the recently diagnosed participants, and young people. Family members were also interested in pension rights, social security entitlements, way to enter job protected and disadvantaged categories, and devices to improve or maintain the independence of MS patients; MS patients did not mention them at all. However, other sources of information can be more useful in case the user did not know exactly what to search for: “I did not know that he was entitled to the disability check until the health care professionals from the Institute told me” [Mother of a MS patient]. Interpretation of diagnostic test results (eg, magnetic resonance imaging, cerebrospinal fluid results) were also searched for.

Participants complained about a certain amount of unsatisfied information needs on the Web. While some topics were actually covered but might not be found because of a lack of confidence in Web searching, as information on pregnancy for MS patients or hereditary tests, some were not really known and were therefore not available, for example, information on a definitive cure or on the causes of MS.

A lack of information on alternative therapies, diet, and lifestyle was highlighted; appreciation was expressed by 1 of the MS patients for the useful US MS society list of the drugs currently in trial in the world. MS patients would need information on how to cope with family members, their concerns, and their obsessive behavior, while family members would need information on how to cope with MS patients; however, it was unclear if they actually searched for it.

Websites that greet the user with a depressing definition of MS, presenting the worst outcomes, was mentioned by MS patients as having a negative impact *on emotional or psychological well-being*. Patients needed some optimism in the way information was presented.

### Sources of Information and Criteria to Discern Accurate Information

Neurologists were the most important source of information for many participants but, among the family members of severely disabled MS patients, the general practitioner became the primary referent. The network of friends was also an important source of information as well as organizations offering services to disabled people. Participants were curious about Web-based information but they were also cautious about its quality and trustworthiness, saying they preferred to discuss drugs and therapies with neurologists. The lack of the necessary skills to distinguish between reliable and unreliable information was given as a reason for opting out of Internet use, especially by the less educated MS patients.

Some subjective criteria were indicated to assess information but they were not universally agreed; also instinct has been mentioned as a form of reliability of information assessment: “I trust my gut instinct” [MS patient, focus group]; “I try to avoid exactly that, not to follow my gut instinct” [MS patient, focus group]. The independence of the source from financial and commercial interests was a shared criterion of trustworthiness, although there was no full agreement on how to identify nonindependent sources. For example, advertisement banners were seen by some as a sign of dependence but not by others “as the banners are everywhere.” The presence of links to downloadable documents including bibliographies and laws was another criteria raised during the discussion; it was considered a valuable opportunity to easily access further details and check the sources of information.

MS associations were mainly considered independent, but not all agreed. The institutional source was often cited as a criterion of credibility, especially the official websites, for example AISM, the Italian Health Ministry, hospitals, and health trusts.

Often the trustworthiness was tested through some kind of overlap: “After reading for four times the same information to me it is the casting out nines” [Mother of a MS patient]. If an item was discussed or presented on several trusted websites, or reported by many sources of information such as newspapers or TV, it was likely to be reliable. If information matched what the neurologist said or was known to be true by the searcher it was considered reliable: “I do a statistic of what is said and if this statistic is comforted by doctor’s opinion I think it is reliable” [MS patient, focus group]. The long age of a website or comments from a neurologist were mentioned indicators of reliability, especially if the neurologist was clearly recognizable and affiliation was stated.

In general, it was taken for granted that the “Internet is always up to date” [Husband of a MS patient].

Participants in the online forum stated they only searched for and trusted reliable Web-based information, but they did not specify how they distinguished between reliable or unreliable information. Websites that were not easily accessible, not clear, and where the date of update was missing were rejected or looked at with suspect.

### Web Search Behavior

Often the aim of seeking information on the Web was to prepare for discussions with the neurologist. Sometimes it was used after the visit to better understand what had been said or to collect information on the options presented by the neurologist. In some cases Web-based information was immediately applied to solve practical problems or cope with symptoms. Sometimes the aim of the consultation was to try to relieve the emotional burden, though this was not always achieved.

As time went on, the Web-based searching became less and expectations dropped, and participants became more cautious. There were two types of Internet users, the ones that rely on few reliable websites, which they regularly consulted like a “habit, as well as you read a newspaper,” and the ones that started their search using search engines, especially for occasional needs.

There were people that stopped searching after an initial period of intensive searching and decided to rely solely on their neurologist for information and advice. On the other hand, people that did not accept their disease or were frightened preferred, almost from the beginning, not to read anything related to MS. Over the time some of them accepted their disease, and became less frightened, and began to search the Internet again:

At the beginning, I tell you one thing, you don’t want to know too much. Yes, you have it, stop. Over time you become aware and want to know more and more.

MS patient, focus group

The decision to opt out of Internet use was usually taken soon after the diagnosis but also in later stages when conditions deteriorated. The tendency of MS patients to opt out of Internet use was clearly raised by family members, who felt committed to search on behalf of the ill person, acting as an information filter even if MS patients complained about this type of behavior. Family members also searched for themselves to overcome their feelings of impotence in face of unpredictability and uncertainty of the disease: “Goodness knows what I would do, I cannot do anything so it seems to me I’m able to help her this way.” [Mother of a MS patients]

At the beginning, people searched for information on the disease, its causes, its mechanism of action, symptoms, and therapy. In the years after diagnosis, they looked for ongoing clinical studies, scientific research, news spread by mass media, and social networks.

In searching for information about new drugs and trials, English speaking people believed English-language websites were more up-to-date and useful than Italian ones. The lack of access to scientific articles, mostly written in English language, was considered a barrier to this kind of information. Medical jargon was another strong barrier to information.

### Social Networks

In some cases the participants rejected the use of social networks, as they preferred to share information face-to-face, and also because of privacy concerns. Social networks were more used to read others’ stories and retrieve information, than to share personal information and experience. Social networks were also used to make decisions about the management of their life with MS.

People using social networks trusted them because they involved MS patients who were considered independent from commercial or other interests:

Information are quite secure given the seriousness of the issues. I do not think that in a forum attended by people who have the disease people say useless things or things that are not true.

Husband of a MS patient

Here again, some criteria were applied to assess the trustworthiness. Consistency of the information, consistency with information received from the neurologist, a competent moderator, a clear statement of the forum objectives, and lack of argumentativeness were all applied.

Those who did not use social networks, considered them unreliable since it was impossible to check the identity of people behind the nicknames, “All of these sites where there are threads posted… first of all I do not know who wrote that, because they have all a nickname, are not recognizable people, I do not know if it is true that they have the disease or if they are people who write just to write I mistrust these” [Husband of a MS patient], or because the participants was no scientific background: “If you go on a forum, it happened to me several times, that someone says that this medicine works this other works, but who are you to say it?” [Partner of a MS patient].

Selected forums could become important supportive tools for newly diagnosed young people or, less frequently, their family members. Facebook was sometimes used as a source of information (especially for new controversial intervention, as for examples CCSVI), or as a space for keeping in contact with friends, “where you did not present yourself as an ill person.”

## Discussion

### Principal Findings

The focus group and the online forum draw a new picture of the information needs of MS patients and family members, pointing to topics and issues to be considered to offer good information. As recently showed by a Cochrane review information, it is central to increase disease-related knowledge and it is in part correlated with the decision making process and quality of life [32]. There are two good reasons to catch patients’ information needs. First, to make relevant information available to patients, a research governance strategy bringing together researchers and patients is needed [33]. Second, a definite judgment of treatment effect needs to incorporate patient’s voice into the design of the therapeutic programs [34].

This study shows a wide spectrum of information needs, such as a comprehensive communication of diagnosis, prognosis, and adverse events of treatment, MS causes or risk factors, new drugs, practical and lifestyle-related information.

The use of the Web varied widely according to personal characteristics, health role (MS patient or family member), and time from the diagnosis. There were people who considered Internet useful for collecting information and learning about others’ experiences, also using social networks, while others were cautious and preferred relying on information given by their neurologist. MS patients were mainly “on demand” users, searching on the Web before and/or after seeing the neurologist or when a new therapy or a new risk factor was proposed. MS patients reported changes in information-seeking over time. They searched Internet for information about the disease extensively, but without a planning, soon after diagnosis. Later, during the course of the disease, they changed their attitude and adopted more focused strategies searching answers to specific questions. Some MS patients gave up Internet searches but were still interested in up-to-date news, particularly new treatments and research results. Family members continued to use Internet searching for information that could help, like exercises or diet, and information on legal rights for people with MS.

The use of the Web was also influenced by other media. The CCSVI was an example of the effect of interactions among different media about health care issues: many people started searching information on the Web about CCSVI after watching television programs on it; on the other side, the Web raised the attention to this topic among MS patients, driving also the attention of other media. This process amplified the messages conveyed and the demands about CCSVI. In this controversial case, the Web played an important role, giving the opportunity to access articles, conference presentations, interviews, and providing practical information, such as searching for hospitals that offered CCSVI diagnosis and treatment.

The use of social networks was also very variable. Some considered online forums reliable because they were written by people living with the disease, not driven by commercial or professional interests, and offered information not provided by neurologists or medical websites, such as matters related to practical and life-style-related information. Others did not use social networks, and considered them unreliable because they are written by lay people, preferring face-to-face interactions to share their experience with other people with MS or the neurologist.

Difficulty to recognize reliable information on the Web constituted a critical issue raised by both MS patients and family members, saying they got confused when information on websites they perceived as reliable collided with information given by the neurologists. MS patients and family members were also not confident about which criteria should be used to assess the quality of Web information and their descriptions were often generic.

Neurologists remained the most important source of information for many participants in our study. This finding agrees with the results of a survey on preferred sources of health information in MS patients in the United States [35], where the most trusted source was a physician. Internet use was more common among the respondents to this survey than in our sample: this could be tied to socioeconomic and educational differences and to the different contexts (Unites States, Italy). For example, in Italy the shares for individuals who used the Internet regularly were almost 50% in 2012 and strong gender, age, and territorial inequalities still persisted [15,16].

### Comparison With Other Studies

Even if the results presented here are related only to the Italian setting, the need to have a reliable source of information, the wide spectrum of information needs of patients and family, and how to navigate through the amount of information available in Internet are issues relevant regardless of the country and the culture. A strong wish to get reliable and independent information, particularly Web-based, was reported both by the Italian and Australian MS patients participating to the focus groups and online forum (data submitted for publication). Distinguishing good quality information and deriving usefulness from it were difficult for many of the participants in both studies, mainly because of information overload and contradictory results they found on the Internet. Searching strategies changed over time in response to information needs but neurologists and MS Societies remained the most trusted information sources for decision making by MS patients in the two countries. A Web-based survey conducted among Italian parents of children with rare diseases described their Internet use profile, and explored how Internet use affected their health decisions [36]. Parents participating in the Web-based survey were more likely than MS patients participating in our study to access the Internet daily, and stated that Web information increased their comprehension of the disease and improved its management. However, there were some key differences between the two studies. First, different study designs. Second, a different health role of parents of children affected with rare diseases and adults patients with MS. Third, a likely high quality format of Web health information for children with rare diseases enabling their parents to make Web-based information applicable and meaningful for their personal circumstances.

### Study Limitations

Although the questionnaire used for participants’ selection focused on Internet use, some family members in the focus groups only occasionally used the Web to find MS information. This limited some findings about their Web-searching behavior and about assessing the quality of websites.

People participating in the online forum tended to answer the questions without launching new topics and rarely sharing comments with others. It could be that inviting people to take part in a research project discussing predefined questions limited their interaction, moreover only 1 month of observation could be a too limited time period. In order to learn about people’s behaviors in participating in a forum it could be better to observe their spontaneous posts and questions.

### Implications for Development of the IN-DEEP Web-Based Source of Information

This is a first qualitative step of a more complex research plan, results come out from a selected group of people, some selection bias are possible. Nevertheless the results show a wide spectrum of inputs to be considered in developing Web-based good information for MS patients and families. In the next stages, we will develop and evaluate a model for presenting health information on the Internet making high-quality evidence, primarily derived from the Cochrane reviews, more accessible and meaningful to MS patients and their families. Some points have been discussed, particularly what topics to include and which “research-based” sources. Other implications from these findings refer to the quality of communication that has to be clear, complete, transparent, and updated to enable people using the information and make it applicable and meaningful for their personal circumstances.

The difficulties in assessing and evaluating the quality of Web-based health information suggest also the need for educational tools, as a glossary and sections with methodological information.

Considering that information needs gradually change along the course of the disease, the Web-based IN-DEEP model will be tailored on three levels of information with increasing level of details. The website format reflected preferences for layered information complexity (ie, “the short answer,” “the detailed answer,” “the deep answer”) and a combination of words, numbers, and pictures to explain benefits and adverse events, with additional sections on practical information, research methodology, and personal histories. Personal experiences were considered useful to convey and reinforce the messages and translate them to daily life [37].

## Multimedia Appendix 1

Screening questionnaire.

## Multimedia Appendix 2

Interview guide.

## Multimedia Appendix 3

Online forum questions.

## Abbreviations

AISM: Italian Multiple Sclerosis Association
CCSVI: chronic cerebrospinal venous insufficiency
EDSS: expanded disability status scale
IN-DEEP: INtegrating and Deriving Evidence, Experiences and Preferences
MS: multiple sclerosis
SQ: screening questionnaire
