# Supplementary material for: Web Search Behavior and Information Needs of People With Multiple Sclerosis: Focus Group Study and Analysis of Online Postings
Source: Interact J Med Res. 2014 Jul 24;3(3):e12. doi: 10.2196/ijmr.3034 (PMC4150054; doi:10.2196/ijmr.3034)
Supplement: Supplementary file 1 [file ijmr_v3i3e12_app1.pdf]

APPENDIX 1. SCREENING QUESTIONNAIRE FOR FOCUS GROUPS

- Sex (Male/Female)
- Age
- Country of birth (Italy/Other)
- City of residence
- Marital status (single/never married; married or cohabiting; separated or divorced; widower)
- Education (primary school; diploma; degree; Master's or doctorate; other)
- Work (employee/worker to full-time; worker /part-time worker; unemployed; student; housewife; retired; any other condition)
- Do you work as an health professional? (Yes/No)
- Year of diagnosis
- Do you have regular contact with AISM Italian Multiple Sclerosis Society (AISM) (Yes/No)
- Do you use the internet? (Yes/No) How often?
- Do you use internet to search for information on MS? (Yes/No)
- Have you a Facebook, Twitter, Myspace account? (Yes/No)
- Have you an e-mail address you check regularly? (Yes/No)
- Type of MS (Relapsing/remitting multiple sclerosis; Secondary progressive multiple sclerosis; Primary progressive multiple sclerosis)
- Disability EDSS (0-10)
- Therapy (list of drugs)
