# Supplementary material for: Web Search Behavior and Information Needs of People With Multiple Sclerosis: Focus Group Study and Analysis of Online Postings
Source: Interact J Med Res. 2014 Jul 24;3(3):e12. doi: 10.2196/ijmr.3034 (PMC4150054; doi:10.2196/ijmr.3034)
Supplement: Supplementary file 2 [file ijmr_v3i3e12_app2.pdf]

APPENDIX 2. INTERVIEW GUIDE FOR FOCUS GROUPS

Topics discussed

What kind of information do you need/search for?

What kind of information sources do you use and prefer?

Do you use the internet? How often?

What kind of information do you search for on the web?

Do you have preferred websites?

Do you start your from a search engine?

How do you know if a website is trustworthy?

Which websites do you prefer?

Do you use social networks?

How would you like a website aimed at people with MS?
