# Supplementary material for: Web Search Behavior and Information Needs of People With Multiple Sclerosis: Focus Group Study and Analysis of Online Postings
Source: Interact J Med Res. 2014 Jul 24;3(3):e12. doi: 10.2196/ijmr.3034 (PMC4150054; doi:10.2196/ijmr.3034)
Supplement: Supplementary file 3 [file ijmr_v3i3e12_app3.pdf]

APPENDIX 3. QUESTIONS ON THE ONLINE FORUM

1 – What sources do you use to find information about treatments for multiple sclerosis? Do you use different sources depending on the type of information you are looking for?

2 – What influences your preference for one internet site over another?

3 – Do you think on-line information related to multiple sclerosis is trustworthy? On what basis do you assess the information?

4 – Regarding information on how to manage multiple sclerosis, what particular on-line discussions do you search for? For example, to manage fatigue, pharmaceutical immunotherapies, diet, rehabilitation, others' experience, etc.

5 – Has your method of online searching changed over time? Have your information needs changed according to the disease phase?

- If yes, how?

6 – Tell us about your experience: how do you search the internet for information on multiple sclerosis treatments?

- How do you begin your internet search?
- How do you follow your search in the various sites?
- Do you have sites you visit regularly or to which you refer? Which ones and why?

7 – Do you think that information about MS is particularly difficult or impossible to find on the internet? What information do you consider useful and what does not interest you?

8 – How do you use the MS therapy and treatment information found on the internet?

9 – Do you use social networks? If yes, do you also use them for MS? Do you think they could be used to manage and cope with MS? If yes, why? If no, why?
